# Covalent triazine framework supported non-noble metal nanoparticles with superior activity for catalytic hydrolysis of ammonia borane: from mechanistic study to catalyst design†
†Electronic supplementary information (ESI) available: ^11^B NMR spectra, XRD patterns, results of BET and ICP, XPS spectra, TOF values and activation energies *E*_a_ of the non-noble metal catalysts, time *versus* volume of H_2_, catalytic activities and TEM images of 5% Co/CNT, 3% Co/CNT, 1% Co/CNT, the plot of hydrogen generation rate *versus* the concentration of Co and AB, kinetic isotope effect and TEM image of 5% Co/CTF-1 after reaction. See DOI: 10.1039/c6sc02456d


**DOI:** 10.1039/c6sc02456d

**Published:** 2016-08-30

**Authors:** Zhao Li, Teng He, Lin Liu, Weidong Chen, Miao Zhang, Guotao Wu, Ping Chen

**Affiliations:** a Dalian National Laboratory for Clean Energy , Dalian Institute of Chemical Physics , Chinese Academy of Sciences , Dalian , 116023 , China . Email: heteng@dicp.ac.cn; b University of the Chinese Academy of Sciences , Beijing 100049 , China; c State Key Laboratory of Catalysis and Collaborative Innovation Centre of Chemistry for Energy Materials , Dalian Institute of Chemical Physics , Chinese Academy of Sciences , Dalian , 116023 , China

## Abstract

Development of non-noble metal catalysts with similar activity and stability to noble metals is of significant importance in the conversion and utilization of clean energy. The catalytic hydrolysis of ammonia borane (AB) to produce 3 equiv. of H_2_, as an example of where noble metal catalysts significantly outperform their non-noble peers, serves as an excellent test site for the design and optimization of non-noble metal catalysts. Our kinetic isotopic effect measurements reveal, for the first time, that the kinetic key step of the hydrolysis is the activation of H_2_O. Deducibly, a transition metal with an optimal electronic structure that bonds H_2_O and –OH in intermediate strengths would favor the hydrolysis of AB. By employing a covalent triazine framework (CTF), a newly developed porous material capable of donating electrons through the lone pairs on N, the electron densities of nano-sized Co and Ni supported on CTF are markedly increased, as well as their catalytic activities. Specifically, Co/CTF exhibits a total turnover frequency of 42.3 mol_H_2__ mol_Co_^–1^ min^–1^ at room temperature, which is superior to all peer non-noble metal catalysts ever reported and even comparable to some noble metal catalysts.

## Abstract 2

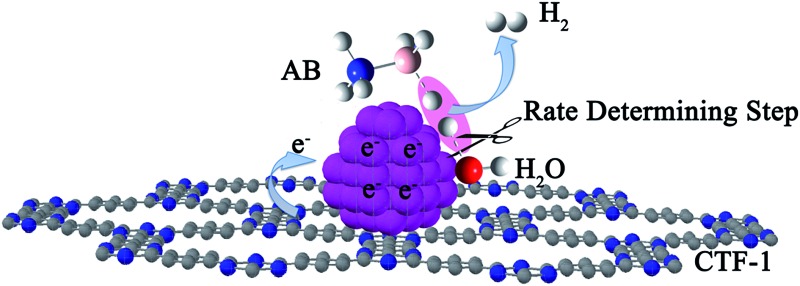
Development of non-noble metal catalysts with similar activity and stability to noble metals is of significant importance in the conversion and utilization of clean energies.

## Introduction

Development of efficient catalysts is of significant importance in the conversion and utilization of clean energies. Usually, the design of catalysts plays a vital role in achieving the desired catalytic properties, which has recently attracted a great deal of interest in terms of structures (size,^1^ morphology^2^ and composition^3^ *etc.*) and supports^4^ of the catalysts. Therefore, the fabrication of novel catalysts with high activity has become a fundamental research front.

Hydrogen, a clean energy carrier, has been widely considered as a potential solution to the energy and environmental crises.^5^ Storing hydrogen in condensed materials with high gravimetric and volumetric densities, proper (de)hydrogenation conditions and/or favorable reversibility, however, remains challenging.^6,7^ Ammonia borane (NH_3_BH_3_, AB for short), with a hydrogen content of *ca.* 19.6 wt%, is a promising hydrogen storage material attracting tremendous research efforts.^8–10^ Hydrogen can be released from AB either through pyrolysis or catalytic hydrolysis (eqn (1)). Although the thermal dehydrogenation temperature for AB is mild, there are drawbacks such as step-wise reaction, sample foaming and toxic byproducts.^11–13^ Catalytic hydrolysis of AB, first introduced by Xu, on the other hand, can generate three equivalents of H_2_ (eqn (1)) rapidly at room temperature.^14–16^ However, the absence of efficient catalysts with low prices made this process still unsuitable for applications.

1 NH_3_BH_3_ + 2H_2_O → NH_4_^+^ + BO_2_^–^ + 3H_2_

Much progress has been made in the development of new structures of catalysts (*i.e.*, amorphous,^17^ nano-particles,^18^ alloyed,^19–21^ core-shelled bimetallic^22–24^ and multimetallic catalysts^25^), which usually possess enhanced catalytic performance in comparison to their crystalline or monometallic counterparts in AB hydrolysis. On the other hand, newly developed functional supports, *i.e.*, metal organic frameworks (MOFs),^26–29^ carbon nanotubes (CNTs),^30^ graphene,^31–33^ carbon nitride,^34^ and organic polymers^35,36^ *etc.*, have exhibited distinct effects on this reaction. For instance, Pt supported on defect-rich CNTs showed superior activity compared to that of pristine and oxygen-doped CNTs due to the electronic modification to the metal by the defects.^37^ MOFs, because of their microporous cages, could confine ultrafine Pt particles, which exhibited extremely high turnover frequency (TOF) for AB hydrolysis.^26^

Although a number of active catalysts have been reported, the development of non-noble metal catalysts with comparative activity as noble metals is still a scientific challenge for this particular reaction. To design and fabricate an efficient catalyst, it is essential to have a clear understanding of the dehydrogenation mechanism, especially the rate determining step (RDS) in the reaction. Previous mechanistic proposals discussed a concerted dissociation of the B–N bond and the hydrolysis of the resulting BH_3_ intermediate.^14,38–40^ The breaking of both B–H and O–H (the slow step) bonds in the process of hydrolysis of AB, on the other hand, was also proposed.^41^ Recently, a theoretical simulation of the hydrolysis of AB on a Ni_2_P surface indicated that an energy barrier of *ca.* 0.12 eV (of AB and H_2_O adsorption) was needed to realize the hydrolysis, in which the adsorption and activation of AB and H_2_O molecules were considered to be the key step for the hydrolysis process.^42^ Although several proposals were put forward, the mechanism observed experimentally is still unclear. Therefore, more experimental work is needed to elucidate the dehydrogenation mechanism, which may further promote the rational design of active catalysts for AB hydrolysis. Herein, kinetic isotope effect (KIE) measurements were conducted to investigate the RDS of AB hydrolysis. Our experimental results demonstrate for the first time that the activation of H_2_O is the RDS in AB hydrolysis. Stimulated by this finding, covalent triazine framework (CTF), a newly developed porous material capable of donating electrons to metals, was selected as a functional support for Co and Ni in the present study, aiming to facilitate the activation of water molecules. To our delight, these catalysts did exhibit remarkably improved activities. Specifically, a TOF of 42.3 mol_H_2__ mol_Co_^–1^ min^–1^ was achieved by the 3% Co/CTF-1 catalyst at room temperature, which is the highest value among all non-noble metal catalysts ever reported and even comparable to some noble metal catalysts.^43^

## Results and discussion

### KIE measurements

**Fig. 1:**
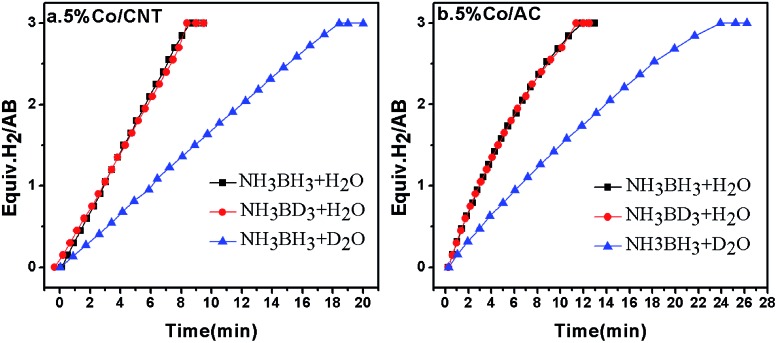
Hydrogen evolution from NH_3_BH_3_ in H_2_O (black), NH_3_BD_3_ in H_2_O (red), and NH_3_BH_3_ in D_2_O (blue) catalyzed by 5% Co/CNT (left) and 5% Co/AC (right) at room temperature, *n*_Co_ : *n*_AB_ = 0.05 : 1.

As mentioned in the introduction section, the RDS for AB hydrolysis needs to be investigated, from which a proper catalyst could be rationally designed. The KIE is considered to be one of the most essential and sensitive tools for the study of the RDS of a reaction.^44,45^ Specifically, it is calculated by the ratio of rate constants for the reactions involving light (*k*_L_) and heavy (*k*_H_) isotopically substituted reactants, *i.e.*, KIE = *k*_L_/*k*_H_, and is classified into primary and secondary kinetic isotope effects.^46^ The primary kinetic isotope effect with a KIE value of 2–7 usually reveals that a bond to the isotopically labelled atom is formed or broken in the RDS. The secondary kinetic isotope effect with a small KIE value of 0.7–1.5, on the other hand, hints that no bond to the isotopically substituted atom in the reactant is broken or formed in the RDS. To shed light on the RDS of AB hydrolysis, KIE measurements were carried out using homemade 5% Co/CNT and 5% Co/AC catalysts. As shown in Fig. 1a, *ca.* 3 equiv. of H_2_ was vigorously generated from NH_3_BH_3_ in H_2_O in 8.3 min catalyzed by 5% Co/CNT. Interestingly, NH_3_BD_3_ in H_2_O exhibited similar dehydrogenation behaviour to that of NH_3_BH_3_ in H_2_O. To exclude the H(H_2_O)–D(BD_3_) exchange during dissolution and reaction, ^11^B nuclear magnetic resonance (NMR) was employed to detect the chemical shift of B in NH_3_BD_3_–H_2_O and in NH_3_BH_3_–H_2_O. As shown in Fig. S1,† no H(H_2_O)–D(BD_3_) exchange was observed during the test. The experimental results above showed that no kinetic isotopic effect occurred when deuteration occurred at the boron (NH_3_BD_3_). However, the hydrolysis of NH_3_BH_3_ in D_2_O showed a slower hydrogen release rate compared to that of NH_3_BH_3_ and NH_3_BD_3_ in H_2_O (Fig. 1). Since H–D exchange can easily occur between D_2_O and [–NH_3_] in AB, the resultant [–ND_*x*_H_3–*x*_] group may also affect the rate of hydrolysis of AB. Actually, since the NH_3_ group does not participate in the hydrolysis, D substitution on the NH_3_ side will have little effect on the reaction rate. Similar KIE results were also observed in the 5% Co/AC catalyzed system (Fig. 1b). The KIE values of 2.1 and 2.2 for Co/CNT and Co/AC were calculated according to the hydrogen generation rates in NH_3_BH_3_–D_2_O systems, respectively, which revealed that the activation of O–H bonds in H_2_O should be involved in the RDS of AB hydrolysis, similar to that of NaBH_4_ hydrolysis.^47^ The involvement of H_2_O activation in the RDS may be partially attributed to its stronger O–H bond strength, *i.e.*, the O–H bond energy (∼493 kJ mol^–1^)^48^ in H_2_O is significantly higher than those of B–N (∼117 kJ mol^–1^) and B–H (∼430 kJ mol^–1^).^49^ In the homogeneous catalytic dehydrogenation of AB, on the other hand, the activation of both B–H and N–H bonds is involved in the RDS as evidenced by KIE investigations.^50^

H_2_O molecules can be activated more easily on noble metals (such as Pt) than on a non-noble metal surface.^51^ This may explain why Pt or other noble metals perform well in water-based reactions, such as the hydrogen evolution reaction^52^ and AB hydrolysis.^30^ H_2_O molecules need to be adsorbed on the metal surface and activated, forming adsorbed –OH and –H species. According to the scaling relations,^53,54^ the adsorption energy of H_2_O on the metal surface should be strong enough to break the O–H bond. The adsorption energies of –OH and –H on the surface, on the other hand, should be intermediate, which will favor the further conversion of these species and liberation of active sites. Therefore, a suitable catalyst for AB hydrolysis should have appropriate adsorption energies for H_2_O, –H and –OH species, which will be discussed and fulfilled in the following sections.

### Catalyst design and synthesis

Since the activation of H_2_O is the RDS for hydrolysis of AB, transition metals that can effectively break the O–H bond in H_2_O to form –H and –OH species of intermediate adsorption strengths are potential catalysts.^41^ In particular, the contribution of support materials to the performance of the final catalysts should be taken into account. The electrochemical hydrogen production from water is usually facilitated by noble metal catalysts which activate water by transferring electrons to the antibonding orbital of a H_2_O molecule.^55,56^ First-principles DFT calculations showed that electron transfer from Nb-doped SrTiO_3_(001) to Pt enhanced its capability in the activation of H_2_O molecules.^57^ Experimental results also indicated that moderately negatively charged Pt^*δ*–^, due to the electron donation from the TiO_2_ nanofiber, performed better with respect to hydrogen evolution from O–H splitting in alcohol.^58^ It is worth mentioning that the negatively charged metal catalyst, derived from the electron donation from the support, is in favor of breaking C–H and N–H bonds as well. For instance, Ru on an N-containing carbon support exhibited higher hydrogen productivity from ammonia decomposition, which was attributed to electron donation from the support to Ru.^59,60^ Similarly, Pt species on N-modified TiO_2_ with high electron density were beneficial to the activation of C–H bonds.^61^ Therefore, it is reasonable to deduce that by increasing the electron densities of non-noble metals, such as Co and Ni, the catalytic performances in AB hydrolysis could be significantly improved.

A number of investigations disclosed that metal nanoparticles supported on nitrogen modified materials could receive electrons from the support leading to enhanced catalytic performance.^62–64^ Theoretical calculation also disclosed the electron donor states of N-doped CNTs to metal particles.^65^ Besides the electron donation effect, the N-doped supports can also provide anchoring sites for metal particles, leading to a good dispersion of catalyst^66,67^ which facilitates the adsorption of reactant or desorption of products.^68^ However, special treatment is required for the preparation of N-doped carbon materials, for which the N-content and the N–C(metal) bonding are poorly defined.^31,63,69^ The newly developed covalent triazine frameworks (CTF) by Thomas *et al.*, on the other hand, possess a number of merits including high surface area, well-defined porous structure and high nitrogen content of *ca.* 17% and thus can be an ideal support material meeting the requirements mentioned above.^70,71^ Recent reports discussed the superior performance of CTF over other carbon-based materials as catalyst supports in oxidation reactions^72–74^ and hydrogenation/dehydrogenation reactions.^75^ Herein, we purposely employ CTF-1, a kind of CTF with a microporous structure (pore size of 1.5 nm) and a surface area around 700–900 m^2^ g^–1^, as the support to enhance the activity of non-noble metal-based catalysts for hydrolysis of AB.

The as-prepared CTF-1 showed a certain degree of crystallization with two diffraction peaks at around 7.3° and 26.1° corresponding to the (100) and (001) planes (Fig. S2†).^70^ The 5% Co/CTF-1 catalyst was synthesized through an impregnation method following the same procedure as for the Co/AC and Co/CNT catalysts. After calcination and reduction, the characteristic diffraction peaks of CFT-1 were maintained in the Co/CTF-1 catalyst. Diffraction peaks belonging to metallic Co or Co compounds cannot be observed in the CTF-1, AC and CNT supported catalysts (Fig. S2†), indicating the fine dispersion of nanoparticles on the supports.

**Fig. 2:**
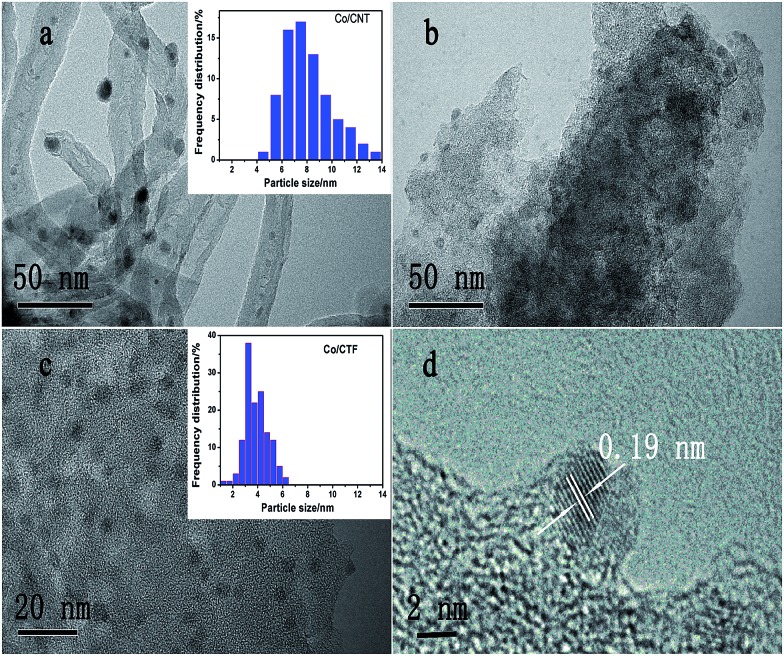
TEM images and corresponding size histograms of (a) 5% Co/CNT, (b) 5% Co/AC, (c) 5% Co/CTF-1, (d) high resolution image of Co particles on CTF-1. The average size is calculated from at least 100 nanoparticles.

Transmission electron microscopy (TEM) images of the three samples showed that all the Co particles are well dispersed in the nanoscale on the supports (Fig. 2), agreeing well with the X-ray diffraction (XRD) measurements. The statistical analysis of the particle size resulted in a mean size of 7.3 nm with a wide distribution for the Co/CNT catalyst (Fig. 2a and Table S1†). Although the size of Co particles distributed on AC was hard to calculate due to the ambiguous boundary, an average size of less than 5 nm was estimated. However, the CTF-1 supported Co has a much smaller mean particle size (3.3 nm) with a narrow diameter distribution. A high resolution TEM image of Co/CTF-1 catalyst in Fig. 2d showed that the interplane spacing of the particle lattice was 0.19 nm, in good agreement with the {101} lattice spacing (0.191 nm) of face centered cubic (fcc) Co, which confirmed that metallic Co was formed after H_2_ reduction. Since CTF-1 is a microporous covalent structure with a pore size of *ca.* 1.5 nm, most of the Co nanoparticles (NPs) on CTF-1 should not be in the pores. Furthermore, considering the hydrophobic property of CTF-1, Co^2+^ solution may not enter the micropores easily. It is worth mentioning that there are 6 N atoms at each opening of the CTF-1 pores, which can serve as anchoring sites for the metal NPs as also pointed out in the literature.^75–77^ We deduce that the Co NPs may mainly stay on the edges of the support and/or the openings of the pores, which blocks some channels of CTF-1 consistent with a significant decrease of surface area from 947 to 726 m^2^ g^–1^ after Co loading (see Table S1†).

### Catalytic performance of Co/CTF-1

**Fig. 3:**
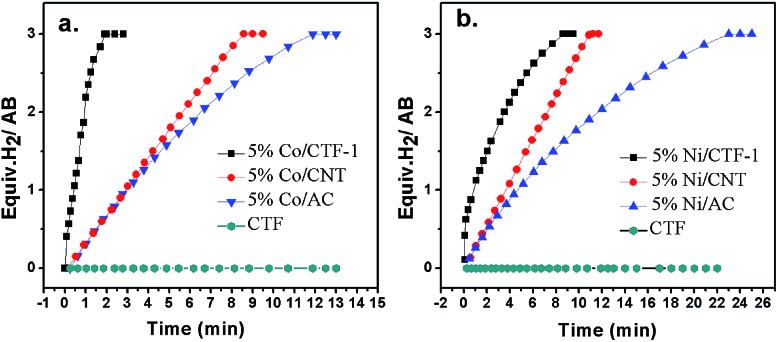
Plots of volume of H_2_ generated *vs.* time for AB hydrolysis catalyzed by (a) 5% Co/CTF-1, 5% Co/CNT, 5% Co/AC, CTF (b) 5% Ni/CTF-1, 5% Ni/CNT, 5% Ni/AC, CTF. ([AB] = 322 mM, 5 mL, *n*_metal_/*n*_AB_ = 0.05).

To check the promoting effect of CTF-1 to Co, the catalytic properties of 5% Co/CTF-1 were tested for the hydrolytic dehydrogenation of AB and compared to those of Co/AC and Co/CNT as shown in Fig. 3. TOFs were calculated to compare to those in the literature, which were based on the real metal content determined by ICP (Table S1†) following the common methods in the literature.^26,31,37^ As mentioned above, the 5% Co/AC and 5% Co/CNT catalysts exhibited moderate catalytic activities, *i.e.*, *ca.* 12 and 8.3 min were needed for each catalyst to complete the dehydrogenation with TOFs of 5.8 and 8.5 mol_H_2__ mol_Co_^–1^ min^–1^, respectively. As we expected, the 5% Co/CTF-1 catalyst showed a remarkably enhanced activity for AB hydrolysis, *i.e.*, only ∼2.0 min were needed with a total TOF value of 33.5 mol_H_2__ mol_Co_^–1^ min^–1^ which was nearly 6 and 4 times as great as those of Co/AC and Co/CNT, respectively. On further decreasing the Co loading to 3%, the TOF of Co/CFT-1 can reach as high as 42.3 mol_H_2__ mol_Co_^–1^ min^–1^, that is among the top most active non-noble metal catalysts, if not the highest, ever reported (Table S2†) and even comparable to some noble metal catalysts.^43^ In addition, a CTF-1 supported Ni catalyst also showed a significantly enhanced catalytic activity as compared to Ni/AC and Ni/CNT (Fig. 3b), suggesting a general promoting effect of CTF-1. It should be noted that the neat support (CTF-1) had no detectable ability in catalyzing the hydrolytic reaction (Fig. 3).

**Fig. 4:**
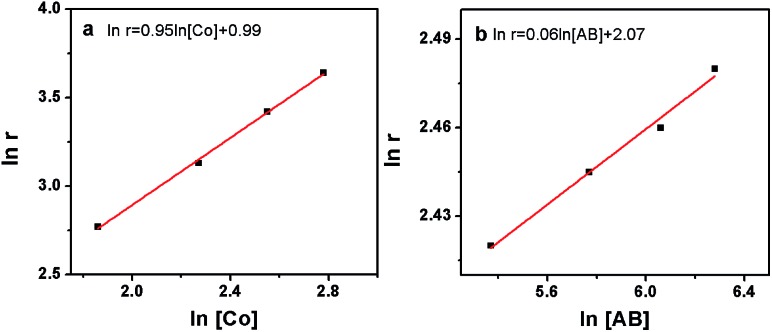
(a) The plot of hydrogen generation rate *versus* the concentration of Co both in natural logarithmic scale, ln(rate) = 0.95 ln[Co] + 0.99; (b) the plot of hydrogen generation rate *versus* the concentration of AB both in natural logarithmic scale, ln(rate) = 0.06 ln[AB] + 2.07.

The kinetics of AB hydrolysis with different concentrations of catalyst and substrate provide valuable information. Fig. S3† shows the plots of volume of H_2_ generated *versus* time for AB hydrolysis catalyzed by the Co/CTF-1 catalyst with different catalyst and substrate concentrations, where almost linear H_2_ gas evolution was observed. The hydrogen generation rates *versus* the concentration of Co and AB, both in natural logarithmic scale, were plotted in Fig. 4. The slope of 0.95 of ln(rate) *versus* ln[Co] indicated that the hydrolysis of AB catalyzed by the Co/CTF-1 catalyst was first-order with respect to the catalyst concentration, which is consistent with the literature.^18^ However, the slope of 0.06 of ln(rate) *versus* ln[AB] indicated that the hydrolysis catalyzed by the Co/CTF-1 catalyst was zero-order with respect to the concentration of AB (Fig. 4b), which implies that AB is easy to activate. Therefore, the activation of AB should not be involved in the RDS, which was in accordance with our KIE results. The kinetic order of H_2_O was also measured in a dimethoxymethane (glyme) solution. As shown in Fig. S4,† the slope of 1.27 of ln(rate) *versus* ln[H_2_O] indicates that the hydrolysis of AB was first-order with respect to H_2_O concentration.

A KIE value of 2.8 was determined for deuteration at H_2_O(D_2_O) for the Co/CTF-1 catalyst, while no KIE was found for deuteration at boron (NH_3_BD_3_) (Fig. S5†), suggesting a similar reaction pathway as the Co/CNT and Co/AC catalyzed reaction where the activation of the O–H bond was still the RDS for the AB hydrolysis reaction.

**Fig. 5:**
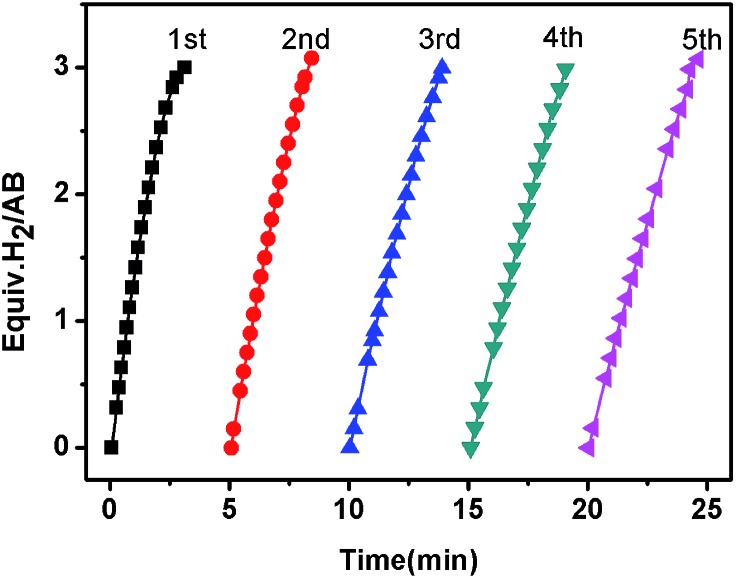
The plots of volume of H_2_ *vs.* time for hydrolysis of AB catalyzed by the 5% Co/CTF-1 catalyst during a five-cycle reusability test.

The reusability of the as-synthesized catalyst under the current room-temperature conditions is critical for practical applications. As shown in Fig. 5, the activity of the Co/CTF-1 catalyst was essentially retained after 5 cycles although a slight drop in reaction rate was observed, which may be attributed to (1) the diluted reactant in water; (2) the increase of solution viscidity with the increasing number of test cycles;^78^ (3) the catalyst surface becoming covered with boron species. Furthermore, TEM images (Fig. S6†) showed that the particle size of Co gradually increased during the tests, which may be caused by aggregation or the deposited borate layer on Co NPs.^18,30^ Nevertheless, the catalysts can be easily separated from the reaction system and recycled following Xu's method^79^ due to the magnetism of cobalt (Fig. S7†).

## Discussion

**Fig. 6:**
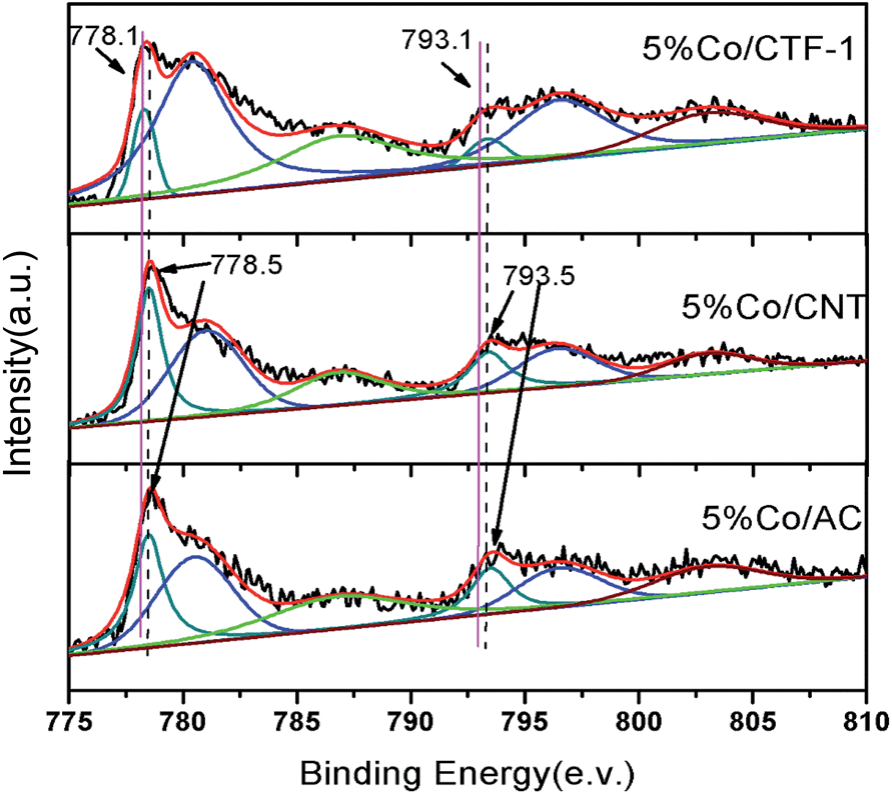
XPS spectra of Co 2p_3/2_ and 2p_1/2_ in 5% Co/CTF-1, 5% Co/CNT and 5% Co/AC samples. All samples were pre-treated with Ar^+^ sputtering.

As mentioned above, the better dispersion of Co on CTF-1 than that of Co on AC and CNT, which can probably be ascribed to the anchoring effect of N on the pore rim of CTF-1,^75–77^ could be one of the reasons for the better activity of Co/CTF. To exclude the particle size effect, Co/CNT catalysts with lower loadings, *i.e.*, 1% and 3%, were synthesized, aiming to obtain similar particle sizes to those of Co on CTF-1. As can be seen from Fig. S8,† the 1% Co/CNT had an average particle size of 3.7 nm which was comparable to that of 5% Co/CTF-1. However, the catalytic activity (TOF of 18.8 mol_H_2__ mol_Co_^–1^ min^–1^) (Fig. S9†) was much lower than that of 5% Co/CTF-1 (33.5 mol_H_2__ mol_Co_^–1^ min^–1^). Therefore, the better activity of Co/CTF-1 should be mainly related to the electronic property of Co. As CTF-1 is an electron donating support, we suspected that Co on CTF-1 would be electron-rich, which would be in favour of H_2_O activation. To confirm the electron transfer between Co NPs and the supports (CTF-1, AC and CNT), X-ray photoelectron spectroscopy (XPS) measurements with and without Ar sputtering were performed (Fig. S10† and 6). Before Ar sputtering, all the Co signals (supported by CTF-1, AC and CNT) can be resolved into two spin–orbit pairs with 2p_3/2_ and 2p_1/2_ binding energies, respectively (Fig. S10†). The binding energies at 780.7 and 796.5 eV can be attributed to Co^2+^, and at 787.2 and 803.0 eV to Co^3+^ species, respectively (Fig. S10†).^80,81^ The formation of Co^2+^ and Co^3+^ species was probably caused by air oxidation during sample loading into the XPS chamber. After Ar^+^ sputtering, a new spin–orbit pair with 2p_3/2_ and 2p_1/2_ binding energies assignable to metallic Co can be detected (Fig. 6),^79^ which was in agreement with the high resolution TEM result. As we expected, the binding energy for Co 2p in Co/CTF-1 had a downshift of ∼0.4 eV compared to that of Co/AC and Co/CNT (Fig. 6), indicating an electron-rich state of Co on CTF-1, which also reflected electron transfer from CTF-1 to Co. As we mentioned in the catalyst design section, electron rich metals benefit several catalytic reactions, including the activation of N–H, C–H, and O–H bonds.^57,59,61^ In accordance with the previous investigations mentioned above, we propose that such an electron-rich state would benefit the electron transfer from Co to the antibonding orbital of H_2_O molecules, resulting in effective activation of H_2_O. Therefore, the negatively charged Co on CTF may favour the activation of H_2_O molecules.

**Fig. 7:**
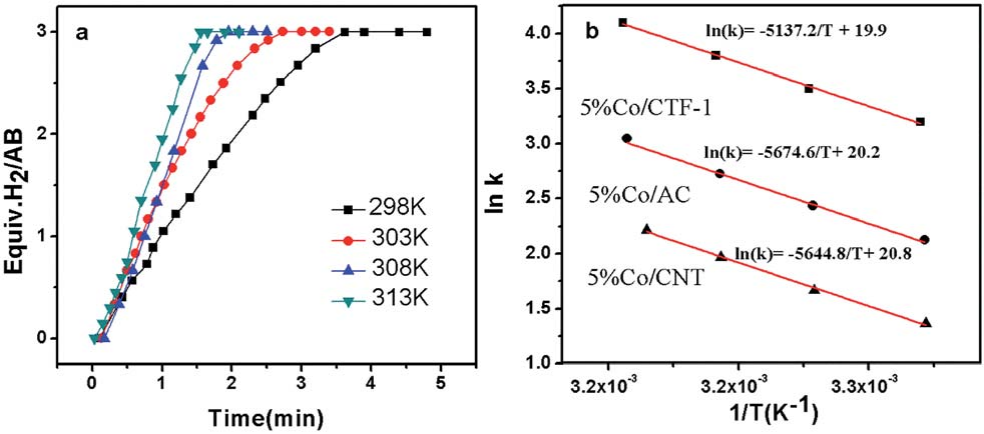
(a) Plots of volume of H_2_ *vs.* time for the 5% Co/CTF-1 catalyzed hydrolysis of AB at temperatures in the range of 298–313 K, catalyst/AB = 0.05; (b) Arrhenius plots obtained from the kinetic data of 5% Co/CTF-1, 5% Co/CNT, and 5% Co/AC.

As H_2_O activation correlates with the electronic structure of transition metals,^51,82^ the apparent activation energy (*E*_a_) of Co/CFT-1 would be reduced compared to Co/CNT or Co/AC. To confirm this, the time dependence of H_2_ generation at different temperatures was recorded (illustrated in Fig. 7) to determine the *E*_a_ through the Arrhenius equation (eqn (2)).2
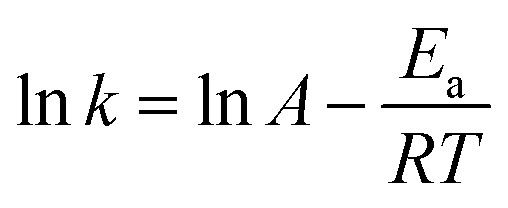



**Scheme 1:**
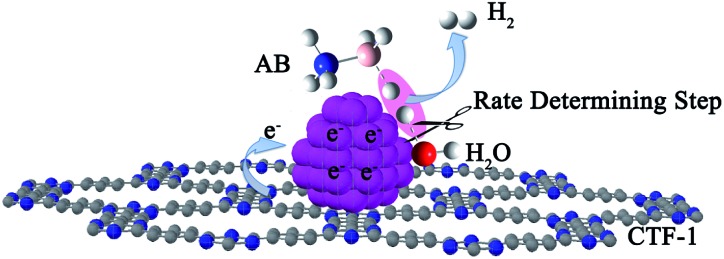
Proposed mechanism for the catalytic hydrolysis of AB by Co/CTF-1.

Fig. 7b shows the Arrhenius plot of ln *k vs.* 1/*T*, from which *E*_a_ can be calculated to be 42.7 kJ mol^–1^ for the Co/CTF-1 catalyst, and 46.9 and 47.2 kJ mol^–1^ for Co/AC and Co/CNT, respectively. The *ca.* 10% decrease in *E*_a_ for the Co/CTF-1 catalyst suggested a favourable transition state with a lower energy barrier, which may be ascribed to the fact that the electron-rich Co was beneficial for the activation of H_2_O molecules. In contrast to the acid catalyzed hydrolysis of AB, where the H^+^ either attacks NH_3_ or BH_3_,^83,84^ the activation of H_2_O and the following steps in our case should occur on the surface of Co.^85^ It is likely that homolytic activation of H_2_O may take place giving rise to –H and –OH, which further react with activated AB to form B–OH and H_2_.^41^ A schematic drawing showing catalytic AB hydrolysis on Co/CTF-1 is presented (see Scheme 1). Owing to the abundance of N in the CTF support, its lone pair electrons may donate partially to Co particles, leading to an electron rich state of Co (as also evidenced by the XPS characterization), which is beneficial to the activation of H_2_O, the rate determining step in the hydrolytic reaction. As a consequence, significantly improved catalytic activity, 42.3 mol_H_2__ mol_Co_^–1^ min^–1^ for the 3% Co/CTF-1 catalyst, can be achieved.

## Conclusions

The KIE measurements show that the RDS for AB hydrolysis is the breaking of an O–H bond in H_2_O. Transition metals with an optimal electron structure that bonds H_2_O and –OH in intermediate strengths may facilitate the hydrolysis of AB. Rich in N, the newly developed CTF-1 has an electron donating effect that markedly increases the electron density of the Co or Ni supported on it. As a consequence, significantly enhanced catalytic activity in AB hydrolysis has been achieved. Specifically, the 3% Co/CTF-1 catalyst exhibited an activity (TOF of 42.3 mol_H_2__ mol_Co_^–1^ min^–1^) that is higher than ever reported for its peer non-noble metal catalysts and even comparable to some noble metal catalysts. The results reported here may pave the way for the rational design of even more highly active catalysts for AB hydrolysis and other reactions.

## Experimental section

### Materials

Terephthalonitrile (liquid chromatogram, Merck), zinc chloride (ZnCl_2_, 98%, Aldrich), ammonium carbonate ((NH_4_)_2_CO_3_, analysis, Acros), sodium borohydride (NaBH_4_, 98%, Aldrich), cobalt nitrate (Co(NO_3_)_2_, analysis, Kermel), nickel nitrate (Ni(NO_3_)_2_, analysis, Kermel), activated charcoal (AC, 20–40 mesh, Aldrich), carbon nanotube (CNT, SMWNT-1020, Shenzhen nanotech port. Co.), ethanol (analysis, Damao) and deuterium oxide (99%, Aldrich) were used without further purification. AB, NH_3_BD_3_ and CTF-1 were synthesized according previous reports.^70,86,87^

### Preparation of catalysts

5% Co/AC and 5% Co/CNT catalysts were prepared by incipient wetness impregnation with ethanol solutions of Co(NO_3_)_2_ and Ni(NO_3_)_2_, respectively. Briefly, the Co^2+^ or Ni^2+^ solutions were dropped into supports uniformly. After ultra-sonication for 20 min, the mixtures were dried at 50 °C for 6 h then 100 °C for 12 h. Subsequently, they were calcined under an argon atmosphere at 350 °C for 4 h. Then, the samples were reduced under a H_2_ atmosphere with a flow rate of 40 mL min^–1^ at 400 °C for 4 h. Finally, the catalysts were transferred under the protection of Ar to the MBRAUN glove box to avoid oxidation or contamination. The same procedure was employed to prepare 5% Co/CTF-1, 3% Co/CTF-1, 3% Co/CNT, 1% Co/CNT, 5% Ni/CNT, 5% Ni/AC and 5% Ni/CTF-1 catalysts.

### Catalytic hydrolysis of AB

The as-prepared catalyst was dispersed into 2 mL of deionized (DI) water in a three-necked round-bottom flask. After that, 0.05 g AB dissolved in 3 mL DI water was injected into the catalyst suspension with a syringe under vigorous stirring. The reaction was carried out at room temperature. The evolution of gas was monitored using a gas buret. In order to investigate the reactivity kinetics, the effect of reaction temperature was studied at different temperatures (298 K, 303 K, 308 K, 313 K) for the hydrolysis of 322 mM NH_3_BH_3_ catalyzed by 16 mM Co/CTF-1. The kinetics order of H_2_O was measured in 5 mL dimethoxymethane with different concentrations of H_2_O (1161 mM, 950 mM, 739 mM, 528 mM) by using the 5% Co/CNT catalyst. TOFs were calculated on the basis of the moles of total metal, following the common methods in literature, where the metal dispersion was not calculated. In fact, the TOF of the catalyst will increase if metal dispersion is considered. All the hydrolysis experiments were monitored in the same way. After the hydrolysis reaction was exhaustively completed, another new batch of NH_3_BH_3_ dissolved in 2 mL of DI water was injected into the solution for testing the recycling stability of the catalyst. The same process was repeated 4 times. All of the catalyst transferring, loading and hydrolysis reactions were conducted under an argon atmosphere to avoid any oxidation and contamination.

### Characterization

TEM (JEM-2100F operating at 200 kV) was used to observe the morphology of catalysts. XRD data were collected on a PAN-alytical X'pert diffractometer equipped with Cu Kα radiation (40 kV, 40 mA). NMR experiments were performed on a Bruker AVANCE 500 MHz NMR spectrometer (11.7 T) at room temperature with the reference to BF_3_ ethyl ether solution at 0 ppm. The specific surface area was measured on an Autosorb-1 system (Quantachrome, USA) by a N_2_ adsorption isotherm through the BET method. The actual Co loadings of the prepared catalysts were determined using ICP spectrometry (ICP-OES, optima 7300DV, Perkin-Elmer, USA). The XPS measurements were performed using an Escalab 250 Xi X-ray photoelectron spectrometer (Thermo Scientific) with nonmonochromatic Al Kα radiation (photon energy, 1486.6 eV), where the sample loading was conducted in air, which may lead to surface oxidization. In order to remove the oxidation layers, the Ar sputtering experiments were performed at a background vacuum of 3.4 × 10^–6^ Pa, a sputtering acceleration voltage of 2 kV, and a sputtering current of 10 mA. Adventitious carbon was used to calibrate the binding energy shifts of the samples (C 1s = 284.8 eV).^88^
