# Supplementary material for: Covalent triazine framework supported non-noble metal nanoparticles with superior activity for catalytic hydrolysis of ammonia borane: from mechanistic study to catalyst design
Source: Chem Sci. 2016 Aug 30;8(1):781–8. doi: 10.1039/c6sc02456d (PMC5299936; doi:10.1039/c6sc02456d)
Supplement: Supplementary file 1 [file SC-008-C6SC02456D-s001.pdf]

Electronic Supplementary Material (ESI) for Chemical Science.
This journal is © The Royal Society of Chemistry 2016

# Supplementary information of

# Covalent triazine framework supported non-noble metal nanoparticles with superior activity for catalytic hydrolysis of ammonia borane: from mechanistic study to catalyst design

Zhao Li[a,b], Teng He[*,a], Lin Liu[a], Weidong Chen[a,b], Miao Zhang[a,b], Guotao Wu[a], Ping Chen[a,c]

[a]*Dalian National Laboratory for Clean Energy, Dalian Institute of Chemical Physics, Chinese Academy of Sciences, Dalian, 116023, China*

[b]*University of the Chinese Academy of Sciences, Beijing 100049, China*

[c]*State Key Laboratory of Catalysis and Collaborative Innovation Center of Chemistry for Energy Materials, Dalian Institute of Chemical Physics, Chinese Academy of Sciences, Dalian, 116023, China*

**Corresponding Author**

E-mail address: heteng@dicp.ac.cn

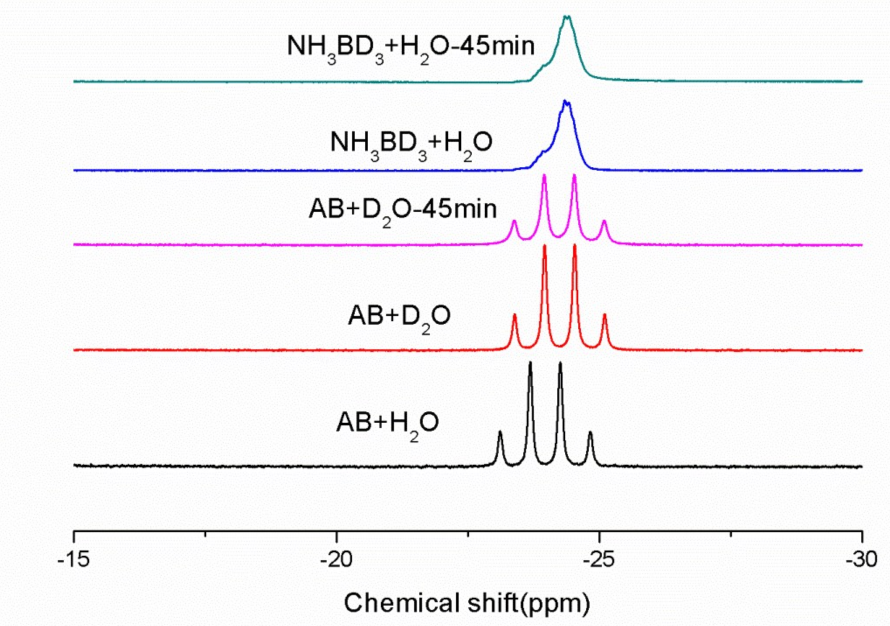


**Figure S1. $^{11}B$ NMR spectra of the AB and $NH_3BD_3$ dissolving in $D_2O$ and $H_2O$ measured at different time.**

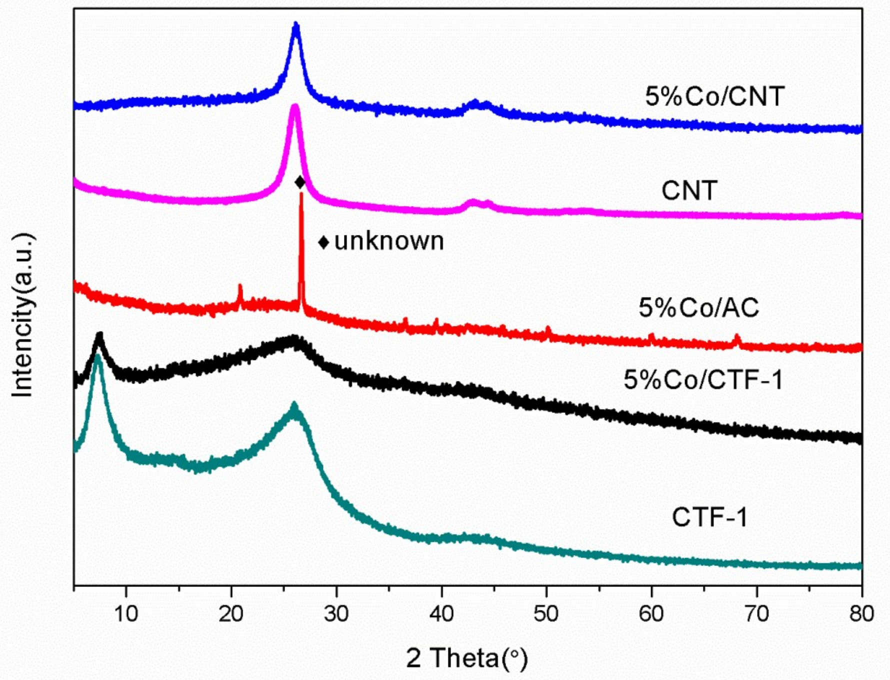


**Figure S2. XRD patterns of CTF-1, CNT, 5%Co/CTF-1, 5%Co/CNT, 5%Co/AC.**

**Table S1. The surface areas before and after loading ($S_{BET}$), Co contents determined by ICP, and average particle sizes of CTF-1, CNT and AC supported catalysts.**

| Catalysts and supports | Co contents (wt%) | Average particle size(nm) | $S_{BET}$ Before loading($m^2/g$) | $S_{BET}$ After loading($m^2/g$) |
|---|---|---|---|---|
| 5%Co/CTF-1 | 4.48 | 3.3 | 947 | 726 |
| 5%Co/CNT | 4.24 | 7.3 | 160 | 156 |
| 5%Co/AC | 4.34 | - | 714 | 685 |
| 3%Co/CTF-1 | 2.83 | - | - | - |

**Table S2. Selective activities in terms of TOF values (mol $H_2$·(mol catalyst)$^{-1}$·min$^{-1}$) and activation energy $E_a$ (kJ/mol) of the non-noble metal catalysts tested in hydrogen generation from the hydrolysis of AB so far.**

| Catalyst | $n_{metal}/n_{AB}$ | TOF | $E_a$ (kJ/mol) | Reference |
|---|---|---|---|---|
| 10wt% Co/ γ -$Al_2O_3$ | 0.018 | 2.30 | 62 | 1 |
| 10 wt %Co/$SiO_2$ | 0.018 | 2.3 | - | 1 |
| 10 wt % Co/C | 0.018 | 2.92 | - | 1 |
| Co/zeolite | 0.02 | 5.36 | 56 | 2 |
| PVP-Co | 0.025 | 4.80 | 46 | 3 |
| PSMA-Co | 0.001 | 25.7 | 34.22 | 4 |
| Co/graphene | 0.05 | 13.8 | 32.75 | 5 |
| G6-OH($Co_{60}$) | 0.013 | 10 | 50.2 | 6 |
| Ni/C | 0.0425 | 8.8 | 28 | 7 |
| 5%Co/CTF-1 | 0.05 | 33.5 | 42.7 | This study |
| 3%Co/CTF-1 | 0.03 | 42.3 | - | This study |
| 5%Co/CNT | 0.05 | 8.5 | 46.9 | This study |
| 5%Co/AC | 0.05 | 5.8 | 47.2 | This study |
| 5%Ni/CTF-1 | 0.05 | 8.75 | - | This study |
| 5%Ni/CNT | 0.05 | 5.4 | - | This study |
| 5%Ni/AC | 0.05 | 2.6 | - | This study |
| Au@Co | 0.02 | 13.7 | - | 8 |
| $Co_{35}Pd_{65}$ | 0.024 | 22.7 | 27.5 | 9 |
| Ag@CoNi/graphene | 0.05 | 15.89 | 36.15 | 10 |
| PEI-GO/Co | 0.11 | 39.9 | 28.2 | 11 |
| $Ni_2P$ | 0.054 | 40.4 | 44.6 | 12 |
| Ni@MCS-30 | 0.016 | 30.7 | - | 13 |
| Pt black | 0.018 | 14 | - | 14 |
| 2 wt.% Pd/γ-$Al_2O_3$ | 0.018 | 1.3 | - | 14 |

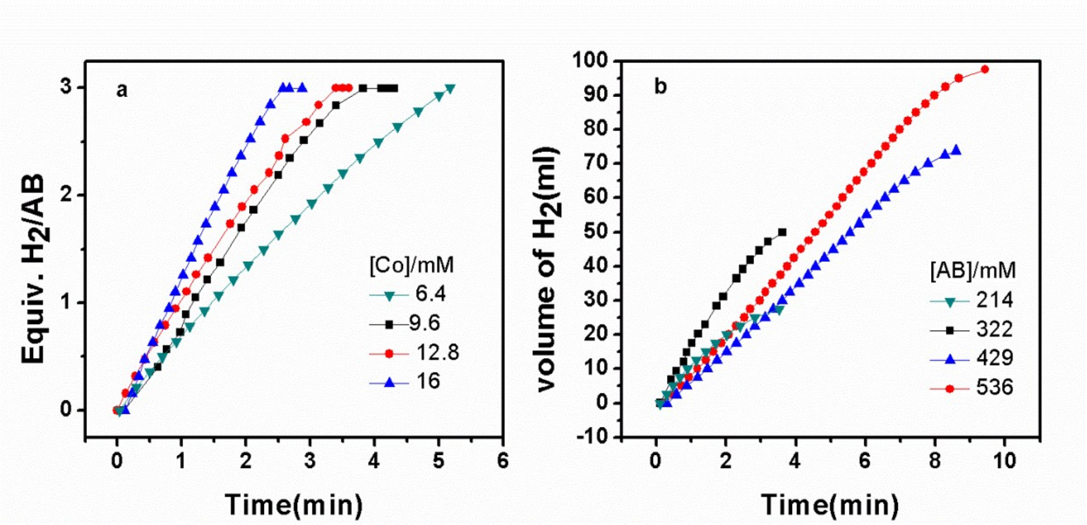


**Figure S3. The Plots of volume of $H_2$ vs. time from AB hydrolysis catalyzed by the Co/CTF-1 catalyst at different catalyst (a) and AB concentrations (b) at T =298 K.**

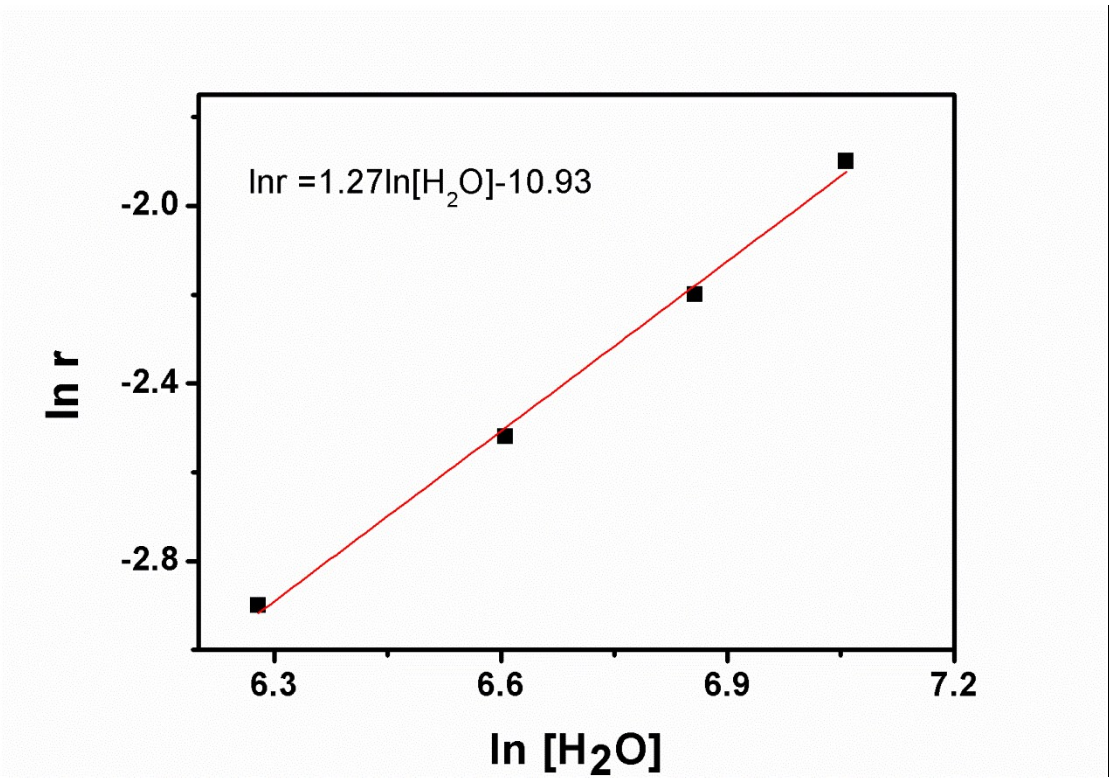


**Figure S4. The plot of hydrogen generation rate versus the concentration of $H_2O$ in natural logarithmic scale, ln(rate) = 1.27 ln[$H_2O$] – 10.93**

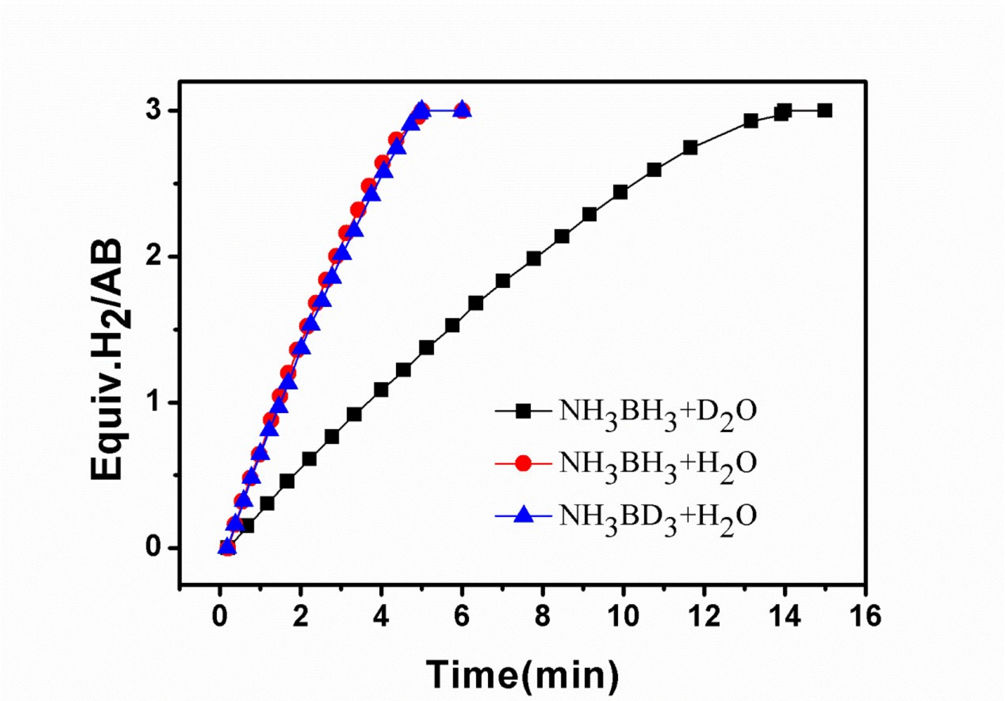


**Figure S5. Kinetic isotope effect of hydrolytic AB catalyzed by 5%Co/CTF-1 at room temperature with $n_{Co}$:$n_{AB}$=0.05:1 for $NH_3BH_3$ in $D_2O$ (black), $NH_3BD_3$ in $H_2O$ (blue) and $NH_3BH_3$ in $H_2O$ (red).**

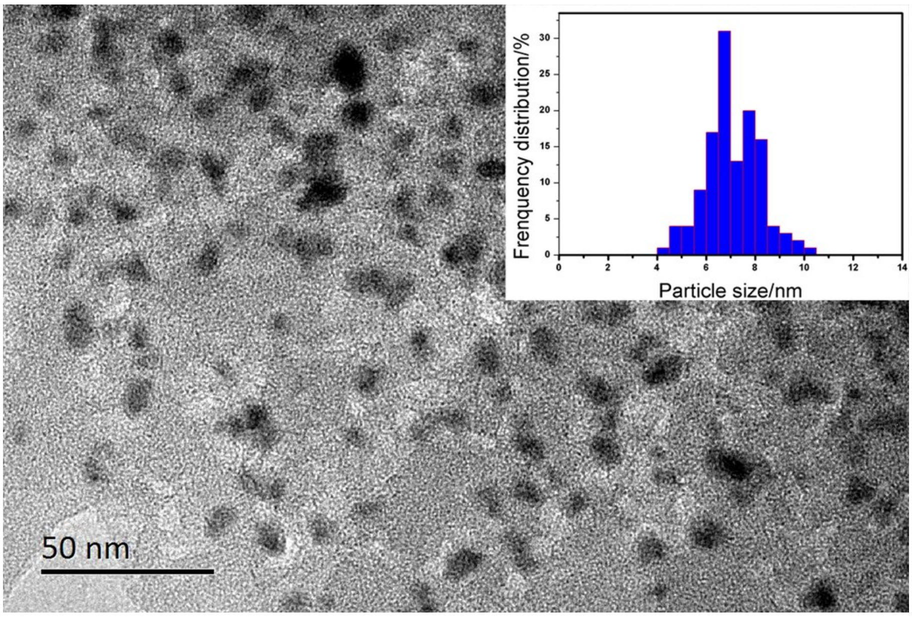


**Figure S6. TEM image and corresponding size histograms of Co/CTF-1 NPs after 5 recycle times.**

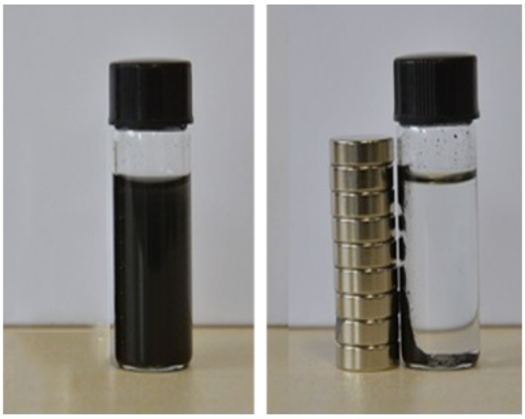

**Figure S7. Left. After hydrolysis reaction; Right. Separation of catalyst by magnet.**

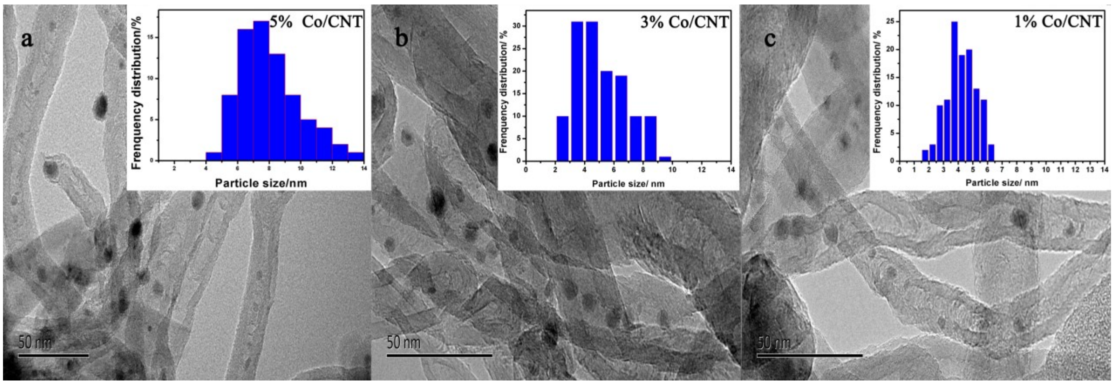


**Figure S8. TEM images and corresponding size histograms of a. 5%Co/CNT, b. 3%Co/CNT, c. 1%Co/CNT. The average size is calculated from at least 100 NPs.**

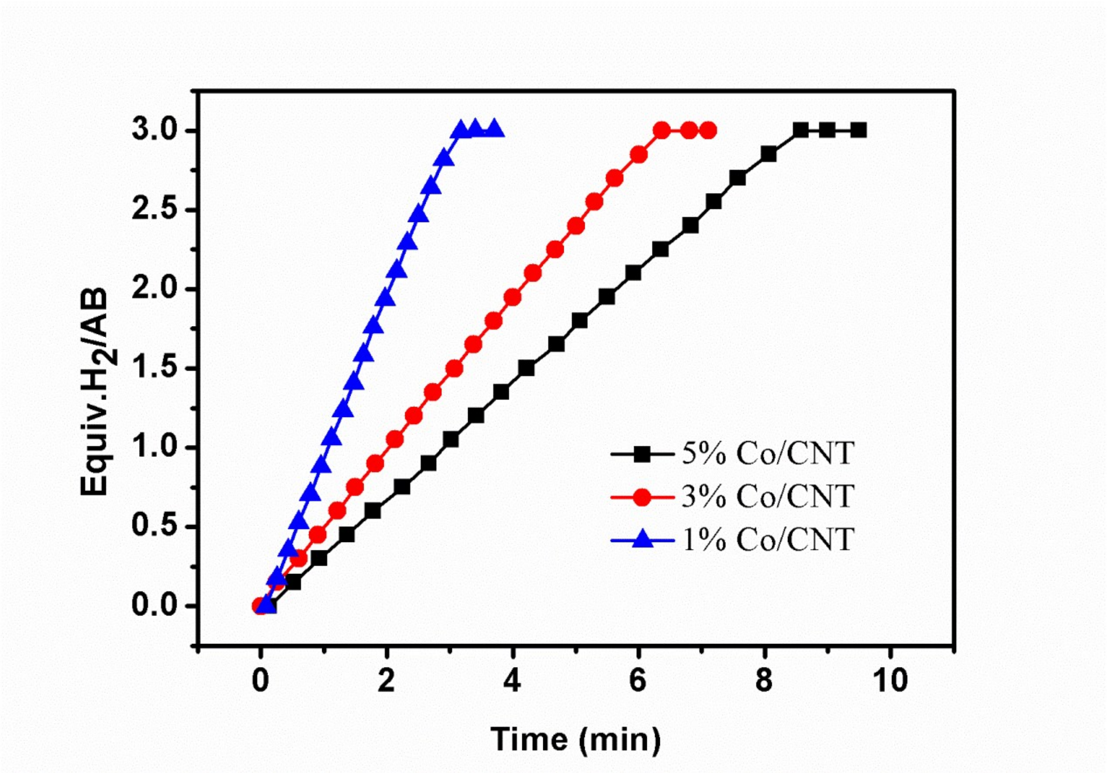


**Figure S9. Plot of time vs volume of $H_2$ generated from AB hydrolysis catalyzed by 5%Co/CNT, 3%Co/CNT, 1%Co/CNT. ( [AB] = 322 mM, 5ml, $n_{metal}/n_{AB}$=0.05)**

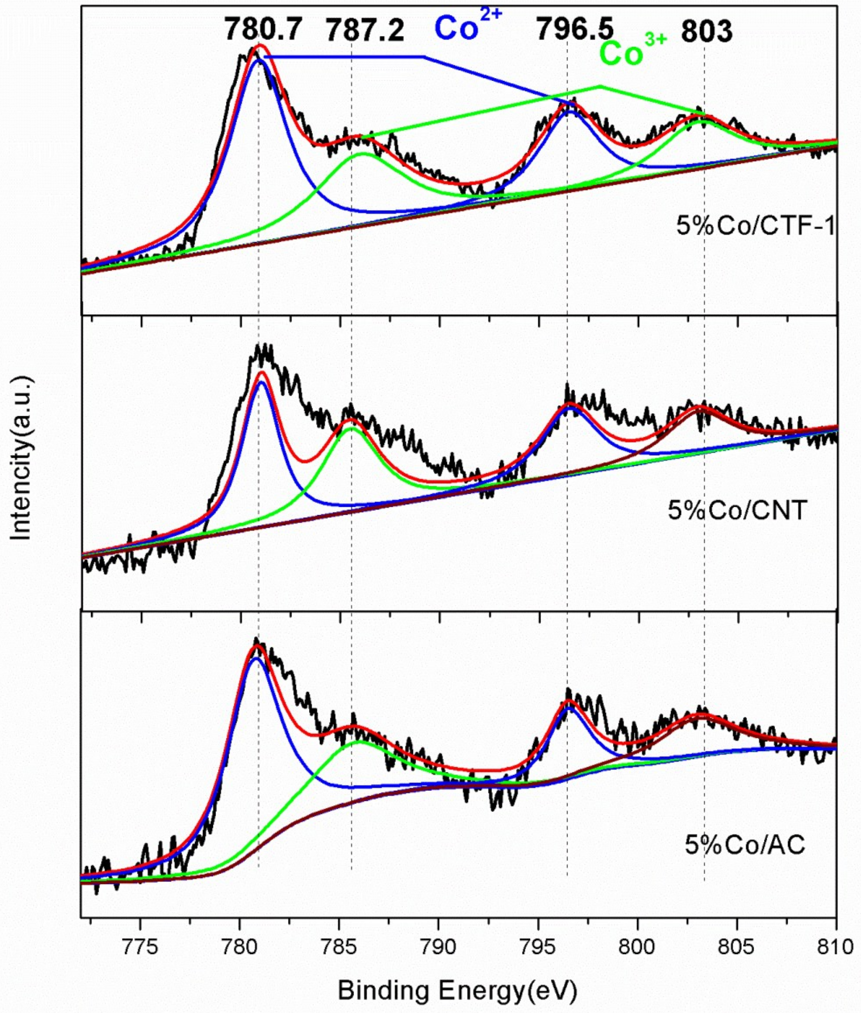


**Figure S10. XPS spectra of 5%Co/CTF, 5%Co/CNT, 5%Co/AC before Ar sputtering.**

**References:**


1. Q. Xu and M. Chandra, *J. Power Sources* 2006, **163**, 364-370.
2. M. Rakap and S. Özkar, *Int. J. Hydrogen Energy* 2010, **35**, 3341-3346.
3. O. Metin and S. Ozkar, *Energy Fuels*, 2009, **23**, 3517-3526.
4. Ö. Metin and S. Özkar, *Int. J. Hydrogen Energy* 2011, **36**, 1424-1432.
5. L. Yang, N. Cao, C. Du, H. Dai, K. Hu, W. Luo and G. Cheng, *Mater. Lett.* , 2014, **115**, 113-116.

6. K. Aranishi, Q. L. Zhu and Q. Xu, *ChemCatChem*, 2014, **6**, 1375-1379.
7. O. Metin, V. Mazumder, S. Ozkar and S. S. Sun, *J. Am. Chem. Soc.* , 2010, **132**, 1468.
8. J.-M. Yan, X.-B. Zhang, T. Akita, M. Haruta and Q. Xu, *J. Am. Chem. Soc.* , 2010, **132**, 5326-5327.
9. D. Sun, V. Mazumder, O. Metin and S. Sun, *ACS Nano*, 2011, **5**, 6458-6464.
10. L. Yang, J. Su, X. Meng, W. Luo and G. Cheng, *J. Mater. Chem. A*, 2013, **1**, 10016-10023.
11. J. Hu, Z. Chen, M. Li, X. Zhou and H. Lu, *ACS Appl. Mat. Interfaces* 2014, **6**, 13191-13200.
12. C. Y. Peng, L. Kang, S. Cao, Y. Chen, Z. S. Lin and W. F. Fu, *Angew. Chem. Int. Ed.*, 2015.
13. P.-Z. Li, A. Aijaz and Q. Xu, *Angew. Chem. Int. Ed.*, 2012, **51**, 6753-6756.
14. Q. Xu and M. Chandra, *J. Alloys Compd.* , 2007, **446–447**, 729-732.
